# Indonesian adolescent girls’ perceptions of electronic sexual-based violence: insights from a qualitative study with clinical expert input

**DOI:** 10.1186/s12889-026-26927-y

**Published:** 2026-03-10

**Authors:** Nurmukaromatis Saleha, Restuning Widiasih, Iqbal Pramukti, Meita Dhamayanti, Yati Afiyanti, Encik Putri Ema Komala

**Affiliations:** 1 Faculty of Medicine, Padjadjaran University, Jalan Ir. Soekarno No. KM. 21, Hegarmanah, Jatinangor District, Sumedang Regency, West Java 45363 Indonesia; 2 Nursing Department, Faculty of Mathematics and Natural Sciences, University of Bengkulu, Jl. Indragiri, Padang Harapan, Gading Cempaka. District, Bengkulu City, Bengkulu 38225 Indonesia; 3 Faculty of Nursing, Padjadjaran University, Jl. Ir. Soekarno KM 21, Hegarmanah, Sumedang, West Java 45363 Indonesia; 4 Department of Child Health, Faculty of Medicine, Universitas Padjadjaran, Sumedang, West Java Indonesia; 5 Faculty or Nursing, University of Indonesia, Jl. Prof. Dr. Bahder Djohan, Pondok Cina, Kecamatan Beji, Kota Depok, Jawa Barat 16424 Indonesia

**Keywords:** Adolescent female, Electronic-based sexual violence, Risk factors, Sex Education, Social Media

## Abstract

**Background:**

Electronic-based sexual violence (ESBV) is an increasingly recognized public health concern, with adolescent girls disproportionately affected, including in Bengkulu, Indonesia. ESBV can be associated with long-term mental health consequences, yet prevention-oriented nursing research in this setting remains limited. This study explored adolescent girls’ perceptions of ESBV and included a clinical psychologist as a key informant to contextualize impacts and recovery needs.

**Methods:**

A qualitative descriptive study was conducted in Bengkulu City, Indonesia. Thirteen adolescent girls from four schools and one clinical psychologist participated in semi-structured, face-to-face interviews in June 2025. Adolescent participants were purposively selected based on teachers’ identification of active social media users. Data were analyzed using thematic analysis. The interview guide was informed by the Health Promotion Model as a sensitizing framework.

**Results:**

Five themes and thirteen subthemes were identified: (1) Gaps in school-based sexuality education beyond biological knowledge; (2) Threats and negative consequences of sexual-social activities in digital spaces; (3) Individual and social vulnerabilities contributing to ESBV risk; (4) Barriers and dynamics of psychological recovery for child and adolescent victims (clinical key informant perspective); and (5) Cross-sector coordination for early self-protection education.

**Conclusion:**

Participants described gaps in school-based sexuality and digital safety education and highlighted the need for protective skills and accessible support. The clinical key informant emphasized barriers to recovery and the importance of coordinated, child-centred responses. Findings may inform school- and family-based prevention initiatives, while broader system-level strategies require further implementation and evaluation research. Future studies should consider longitudinal designs and include male adolescents to support more inclusive prevention approaches.

**Clinical trial registration:**

Not applicable.

## Background

Globally, adolescent girls aged 10–19 years are undergoing a critical developmental transition and face substantial challenges related to their health, sexual rights, and reproductive well-being [1, 2]. One increasingly prominent challenge is Electronic Sexual-Based Violence (ESBV), a form of sexual violence facilitated by digital technologies that has emerged as an unintended consequence of the rapid expansion of social media and online communication platforms. This phenomenon has been recognized as a significant global issue and has received growing attention within the United Nations Sustainable Development Goals (SDGs), particularly in efforts to eliminate violence against women and children, with evidence indicating a steady increase in prevalence worldwide [3–5].

Although Online Child Sexual Abuse (OCSA) and Electronic Sexual-Based Violence (ESBV) are both situated within the broader framework of Technology-Facilitated Sexual Violence and Abuse (TFSVA), this study conceptualizes them as distinct constructs [6–9]. This distinction is grounded in fundamental differences in victim characteristics and underlying power dynamics. ESBV primarily reflects gender-based violence rooted in gender inequality and social control and can affect females across age groups, whereas OCSA is age-specific, centering on children’s developmental vulnerability, in which consent is never legally or ethically valid. By distinguishing between OCSA and ESBV, this study enables a more comprehensive understanding of the intersecting age- and gender-based vulnerabilities experienced by adolescent girls and provides a stronger foundation for the development of targeted nursing and health promotion interventions.

End child prostitution, child pornography and trafficking of Children for sexual purposes (ECPAT) notes that one in three children under the age of 18 in the world is an internet user [10]. Global estimates on the prevalence of child sexual abuse: approximately 1 in 5 girls and 1 in 7 boys experience sexual violence before 18. Systematic evidence further supports a lifetime prevalence of around 19–20% for females and 7–8% for males, indicating substantial gender disparity in victimization during childhood and adolescence [11]. The internet is an integral part of children’s and adolescents’ daily lives, providing access to interpersonal communication, information, and entertainment. Social media has become a primary digital space for social interaction and self-expression, including expressions of sexuality. This environment increases adolescents’ exposure to ESBV, which often remains unrecognized by those affected [12–15]. ESBV can have lasting adverse effects on individuals’ physical and mental well-being [16–18].

In Indonesia, data from the Online Information System for the Protection of Women and Children 2024 indicate that girls are five times more likely to experience electronic sexual-based violence (ESBV) than boys [19]. ESBV refers to sexually violent acts facilitated by information and communication technologies, including non-consensual recording or distribution of sexual content, cyberstalking, and sexual extortion. Although Indonesian legislation has incorporated protective measures such as the right to be forgotten, ESBV extends beyond legal and technological concerns [20, 21]. Its cumulative and developmentally disruptive mental and physical health impacts position ESBV as an emerging public health issue, highlighting the need for comprehensive prevention strategies that integrate sexual health education and nursing-led health promotion.

Nurses play a crucial role in preventing electronic-based sexual violence (ESBV) as researchers, educators, and health promoters, particularly in school and community settings [22]. According to the World Health Organization (2017), universal school-based prevention programs have been shown to be effective and cost-effective in strengthening adolescents’ personal safety skills and reducing their vulnerability to sexual violence [23]. However, these programs often have limited reach due to their reliance on school infrastructure and the limited availability of alternative delivery modalities. Evidence from international studies therefore highlights the importance of introducing comprehensive sex education in schools as early as possible to prevent adolescents from engaging in online sexual risk behaviors and technology-facilitated sexual harm [24–28].

In Indonesia, research on sexual violence against women and children has predominantly focused on offline forms and national-level data, with limited attention to technology-facilitated sexual violence in local contexts. In Bengkulu Province, existing studies have examined child sexual violence, adolescents’ sexual knowledge, and school-based prevention efforts, yet have not addressed electronic sexual-based violence (ESBV) as a distinct phenomenon or explored adolescent girls’ experiences in digital spaces. Furthermore, much of the existing research focuses on legal interventions, with little focus on health interventions, specifically preventive measures [29–32]. This gap is increasingly critical given the growing use of the internet and social media among adolescents and evidence that online sexual victimization is often underreported. Consequently, little is known about how adolescent girls in Bengkulu city perceive online sexual risks, recognize ESBV, or protect themselves in digital environments. This study addresses this gap by exploring adolescent girls’ perceptions of ESBV to inform contextually relevant, school-based nursing and health promotion interventions.

In the Bengkulu context, data from local services and news reports indicate that violence against women and children including sexual violence against children remains a recurring problem [33]. For adolescent girls, online sexual victimization generally occurs through everyday digital interaction patterns: private communication on social media messaging apps/DMs, requests for sexual content, pressure or coaxing (grooming), and threats to spread intimate content (sextortion). These patterns position cyberspace as a “risky situation” that easily integrates with adolescents’ social relationships, thus requiring a contextual understanding before designing school-based prevention. This study is novel in its use of a health promotion framework to explore adolescent girls’ perceptions of ESBV within a local Indonesian context, addressing a gap in context-specific and prevention-oriented evidence.

We initially aimed to include adolescent girls with direct ESBV experiences; however, access to victims could not be obtained because permission was not granted by the Women and Children Protection and Empowerment Service. Therefore, this study explored adolescent girls’ perceptions of ESBV and included a clinical psychologist as a key informant to provide professional insight into victim impact, recovery dynamics, and prevention needs. Specifically, we examined (1) how social media interactions may become risky situations (2), forms of victimization adolescents recognize, and (3) prevention needs perceived as relevant. These findings are intended to inform contextually appropriate promotive and preventive, school-based nursing and health promotion interventions.

## Methods

### Study setting and participants

A qualitative descriptive design that was locally based was used in this study, which was conducted in Bengkulu City from April to June 2025 [34–36]. A qualitative descriptive design was used because it is well-suited for exploring female students’ perceptions of electronic sexual violence in depth and contextually. ESBV is an understudied and sensitive phenomenon. The researcher wanted to obtain direct descriptions of how female students perceive and respond to risks in the digital space, regardless of a rigid theoretical framework. The findings are expected to directly inform the development of school-based nursing interventions and health promotion.

The research team consisted of six healthcare professionals: three maternity nurses, one community nurse, one mental health nurse, and one pediatrician who specializes in child development. None of the research team knew the participants before the research was carried out. These professional backgrounds shaped the research perspective, which focused on a biopsychosocial approach, adolescent development, and efforts to prevent sexual violence. The lead author has a research roadmap and publications related to sexual violence prevention interventions for women of various ages. The researchers recognized that their academic backgrounds could influence the data collection and interpretation processes. Therefore, they implemented continuous reflexivity through team discussions and by deferring initial assumptions to ensure the research findings accurately represented the perceptions of adolescent girls.

The authors presented the results of this study in accordance with the Consolidated Criteria for Reporting Qualitative Research Studies (COREQ) checklist. Data collection took place after the study received approval from the Research Ethics Committee of Padjadjaran University in Bandung (registration number 2502050157, number 271/UN6.KEP/EC/2025). The study also adhered to the Declaration of Helsinki. Written informed consent was obtained from all participants. For participants under the age of 16, written informed consent was obtained from their parents or legal guardians prior to participation. Assent was also obtained from the minors. The researcher provided the clinical psychologist, adolescent female participants, and their parents/guardians with oral and written information in Indonesian about the purpose, procedures, and benefits of the study. Participation was entirely voluntary, and individuals were free to refuse or withdraw at any time. Those who agreed to participate after receiving an explanation signed a consent form. The lead authors kept the form.

To ensure the confidentiality of the participants, the first author provided them with a code known only to the author and stored all data, transcripts, and audio recordings on a personal device. The authors omitted all direct and indirect identifying information and modified all quotations, indicating individual, institutional, and location identification, while maintaining the substantive meaning of the data. The clinical psychologist was included as an expert key informant to provide a professional perspective on victim impact and recovery dynamics, serving as source triangulation to enhance the credibility of findings. Furthermore, a clinical psychologist was involved to provide psychological support and report any participants experiencing psychological distress.

Because ESBV is a sensitive topic, participants were informed that they could pause or withdraw at any time without consequence to minimize psychological discomfort. The interviews were conducted in private areas at the participants’ schools or homes. Interviewers monitored emotional reactions throughout the session and discontinued sensitive inquiries if signs of distress emerged. A collaborating clinical psychologist was available to provide immediate psychological support if needed. A brief debriefing session was conducted at the conclusion of each interview to ensure the participants’ emotional stability. Participants were provided information about available counseling services, including school counseling units, as well as referrals to the collaborating clinical psychologist. The psychologist was appointed by the Women and Children Protection and Empowerment Service, a partner of the office.

Participants were selected through purposive sampling based on the involvement of key individuals, particularly teachers from four schools where cases of ESBV were reported (one junior high school and three senior high schools). Inclusion criteria included: (1) female adolescents aged 13–18 years; (2) enrolled as students at the school; and (3) active on social media. Exclusion criteria included adolescent girls who had been victims of sexual violence. Teachers identified “active social media users” based on observable indicators such as active posting on social media feeds, engagement in online interactions with peers, and known participation in digital platforms commonly used by adolescents. Teachers did not assess content behavior but provided contextual identification based on daily interactions with students. Variation across school background was deliberately incorporated into the sampling strategy by recruiting participants from one junior high school and three senior high/vocational schools representing different socio-educational contexts, age ranges, and academic orientations. This approach enabled the study to capture developmental differences between early adolescents (13–15 years), who are in earlier stages of identity formation and digital socialization, and mid-to-late adolescents (16–18 years), who typically demonstrate greater online autonomy, more complex peer and romantic dynamics, and more advanced risk appraisal capacities. Additionally, differences between general academic and vocational school environments—such as peer interaction patterns, technology use habits, and access to school-based support systems—were considered to explore how developmental stage and institutional context intersect in shaping adolescents’ perceptions of digital risk and self-protection.

The researchers contacted sixteen adolescent girls, thirteen of whom agreed to be interviewed in person. Data saturation was operationally assessed through concurrent data collection and coding. After each interview, transcripts were coded independently by three researchers and compared through constant comparison. A saturation monitoring table was used to document newly identified codes and their frequency across interviews. By the tenth interview, no additional primary codes were generated, and subsequent data were fully accommodated within the existing coding framework. Three further interviews were conducted to confirm code stability; these interviews yielded only repetition and elaboration of previously identified categories without introducing new thematic dimensions. Saturation was therefore determined based on code redundancy, thematic stability, and the absence of emergent conceptual variation across consecutive interviews, indicating analytic sufficiency. Participant characteristics can be seen in Table 1.

**Table 1:** Participant demographic data

Participant Code	Age (years old)	School level	Living together
A1	16	Senior High School	Parent
A2	15	Senior High School	Grandparents
A3	18	Senior High School	Parent
A4	16	Senior High School	Parent
A5	16	Senior High School	Parent
A6	15	Senior high school	Parent
A7	17	Vocational School	Parent
A8	14	Junior high school	Parent
A9	15	Junior high school	Parent
A10	14	Junior high school	Parent
A11	15	Junior high school	Parent
A12	15	Junior high school	Parent
A13	15	Junior high school	Parent
			Length of work
P	36	Magister Psicholog	10 years

### Data collection

The phenomenon of ESBV is explored using semi-structured interviews with a guide modified from the Health Promotion Model. All researchers and participants were fluent in the Indonesian language used in the interviews. The Health Promotion Model (HPM) was used as a sensitizing framework to inform the development of the semi-structured interview guide and to ensure coverage of individual, interpersonal, and situational influences relevant to health-promoting and protective behaviors. HPM was not used to impose predefined categories during data collection; rather, it provided an initial orientation for probing participants’ perceptions of risk, self-protective practices, and contextual influences [37, 38]. In the context of electronically based sexual violence (ESBV), social media serves as a situational influence that increases adolescents’ risk exposure, particularly when risk perception, self-efficacy, and interpersonal support are limited.

The interview guide for adolescent females was: (a) What is your view on the phenomenon of ESBV? (b) Is there a relationship between ESBV behavior and health? (c) How is the prevention effort against ESBV described in your school? (d) Tell us about the sexual violence prevention education in your school? To test the feasibility and validity of the interview guide, the author conducted a trial interview with an adolescent female. Based on the trial results, four questions about dating behavior, getting to know strangers on social media were removed because they could be represented in the second question, so that no context was lost from the objectives that had been made. The trial interview was not included in the main study.

The interview guide for clinical psychologist was: (a) Tell us about your experience in dealing with victims of ESBV cases. (b) How they can become victims of ESBV, (c) Are there differences in children’s vulnerability to KSBE related to gender? (d) What suggestions can you give for preventing ESBV? These questions served as conversation starters, not closed-ended questions, and were developed through flexible follow-up questions (probing questions) based on participant responses. During the interviews, the researcher used probes such as “Can you tell me more?“, “How did you feel at that moment?“, and “How do you usually react to that?” to deepen the narrative, clarify meaning, and explore the context of the participants’ experiences. The interview process was recorded and documented in the form of photos and videos. Each participant underwent one interview session with a duration of between 30 min and 1 h. The interview results were transcribed verbatim after each interview session. Questions and probing can be seen in Table 2.

**Table 2:** List of questions and probing for adolescent girls and clinical psychologists

No	Questions	Probing
Adolescent Females		
1	Tell us about the sexual violence prevention education program at your school.	a. What forms of sexual violence education activities have you encountered at school? (class materials, seminars, guidance counselors, student council, orientation, posters, etc.) b. Who delivered the material? (teachers, guidance counselors, outside parties) c. What material do you remember most? d. Has the school ever discussed ESBV/online harassment specifically?
2	What is your view on the phenomenon of ESBV?	a. What form do you see/hear most often? (comments, DMs, groups, content sharing, etc.) b. Where does it usually occur? (specific platforms, class groups, anonymous accounts) c. In your opinion, who is most vulnerable to ESBV and why? d. What factors contribute to this vulnerability? (romantic relationships, seniority, popularity, peer pressure, etc.)
3	Is there a link between ESBV behavior and health?	a. Does ESBV have an impact on physical and mental health? b. Can it have an impact on academic performance? c. Does the SHU play a role in ESBV prevention education?
4	What are the efforts to prevent ESBV at your school?	a. a. In your opinion, what kind of ESBV prevention measures has your school implemented? b. b. When a case occurs, is there a place to report it? c. c. Do you have any suggestions for ESBV prevention measures at your school?
Clinical Psychologist		
1	Tell us about your experience in dealing with victims of ESBV.	a. Since when have you been handling ESBV cases? b. Are there more child or adolescent victims? c. What form of ESBV is most commonly encountered (e.g., online grooming, dissemination of intimate content, sextortion)? d. What are the most prominent psychological effects on victims? e. Are there differences in psychological responses between child and adolescent victims? f. What are the main challenges you face in the process of assisting ESBV victims?
2	Are there differences in children’s vulnerability to ESBV based on gender?	a. Based on your experience, are girls or boys more vulnerable to becoming victims of ESBV? b. What factors cause this difference in vulnerability? c. Do social norms and gender constructs influence children’s vulnerability? d. How do reporting patterns differ between female and male victims? e. Are boys less likely to disclose experiences of ESBV? f. Are there specific forms of ESBV that are more commonly experienced based on gender?
3	How can they become victims of ESBV?	a. Through which media or platforms do victims most often experience ESBV? b. How does the initial process of victim involvement occur, leading to them ultimately becoming victims? c. Are the perpetrators usually people known to the victims or strangers? d. What risk factors most often arise (e.g., lack of supervision, low digital literacy, need for affection)? e. What role do the school environment and friendships play in ESBV cases?
4	What advice can you give for preventing ESBV?	a. What preventive measures have been most effective in your experience? b. What role do parents play in preventing ESBV? c. What should be taught to children regarding digital safety? d. How can schools contribute to preventing ESBV? e. What role can health workers/psychologists play? f. What key messages need to be conveyed to children and adolescents? g. What policies or support need to be strengthened by the government/relevant agencies?

### Data analysis and trustworthiness

The transcripts were analyzed using thematic analysis following six phases: familiarization with the data, generating initial codes, generating initial themes, reviewing themes, refining/defining/naming themes, and writing up [37]. NS, EK and RW independently read each transcript repeatedly (at least three times) to deepen familiarization and identify preliminary patterns. Relevant meaning units (sentences or paragraphs) were then extracted, condensed, and labeled as initial codes. Codes were compared and grouped based on similarities and differences to develop categories and preliminary themes, which were iteratively reviewed and refined through team discussions involving all authors (NS, RW, IM, MD, YA, EK) until consensus was reached on the final thematic structure.

This analytic process was primarily inductive: coding and theme development were grounded in participants’ accounts rather than derived from a priori Health Promotion Model (HPM) constructs. After themes had been developed, reviewed, and refined, the final thematic structure was subsequently mapped onto relevant HPM components as an interpretive step to support theoretical integration and to inform implications for health promotion practice.

Trustworthiness was addressed through credibility, dependability, confirmability, and transferability procedures [39]. For credibility, analysis was conducted concurrently with data collection; transcripts were coded by multiple researchers (NS, RW, EK) and discrepancies were resolved through consensus meetings (NS, RW, IP, MD, YA). In-interview checking and brief debriefing with summary confirmation were used to ensure alignment with participants’ intended meanings; transcript return was not performed to reduce potential discomfort and emotional reactivation among adolescents. For dependability, we maintained an evolving codebook and decision log, supplemented by a code monitoring table to document when codes ceased to emerge and became repetitive; three additional interviews were undertaken to verify thematic stability. For confirmability, an audit trail (transcripts, field notes, analytic memos, codebook iterations, and peer debriefing notes) was maintained and reflexive discussions were held throughout analysis. Transferability was enhanced through rich description of context and participant characteristics and by presenting verbatim excerpts.

## Results

**Fig. 1:**
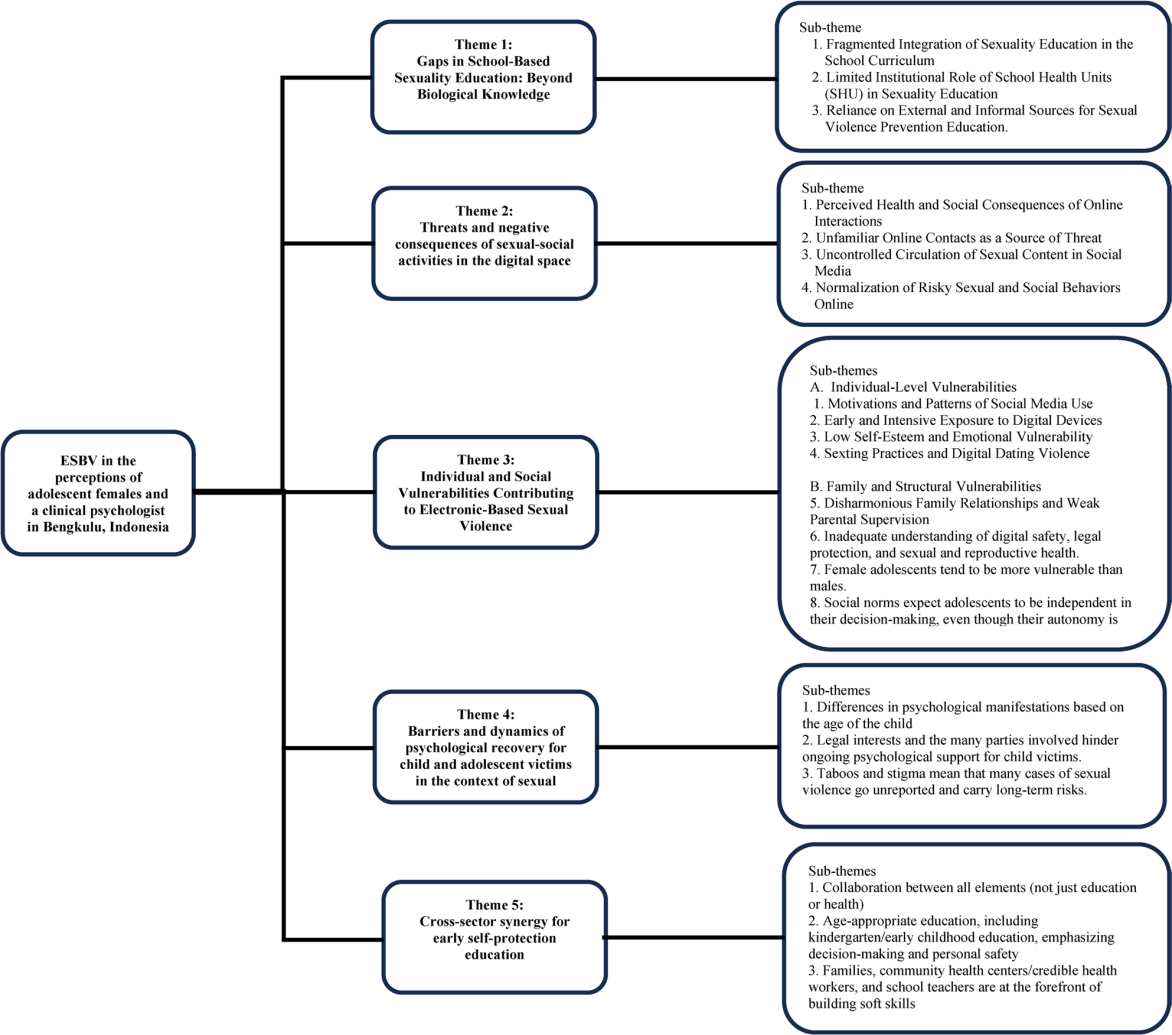
Themes and subthemes

Adolescent females’ and clinical psychologist’ perceptions of the ESBV phenomenon in Bengkulu City can be seen in Fig. 1. Five main themes are:(1) Gaps in School-Based Sexuality Education: Beyond Biological Knowledge; (2) Threats and negative consequences of sexual-social activities in the digital space; (3) Individual and Social Vulnerabilities Contributing to Electronic-Based Sexual; (4) Barriers and dynamics of psychological recovery for child and adolescent victims in the context of sexual violence; (5) Cross-sector synergy for early self-protection education.

### Theme 1: Gaps in school-based sexuality education: beyond biological knowledge

There are three sub-themes, namely:

1. Fragmented Integration of Sexuality Education in the School Curriculum
  Participants’ quotes show a gap between the importance of the school’s role and the practice of self-protection/safe behavior education provided.
  A1: “So far, there hasn’t been any. At most, the teacher would suddenly say, ‘Don’t get close to boys for fear of something bad happening,’ or ‘Just watch good content when playing with your phone”.
  The absence of structured intervention indicates that the delivery of material is incidental, rather than part of planned learning. Normative advice and simple prohibitions tend to be oriented toward restriction and fear, rather than strengthening children’s ability to recognize risks, set boundaries, and seek help. Emphasis is placed on control without building digital literacy.
  A3: “Well, so far, the teachers haven’t really taught them much about these kinds of issues.”
  P: “Because nowadays, school-aged children spend almost all their time, nearly 8 hours, at school… which means that the role of schools is very important in shaping how children behave appropriately, how children have the ability to prevent it, for themselves.”
2. Limited Institutional Role of School Health Units (SHU) in Sexuality Education
  This quote implies that schools may have school health unit (SHU) resources that could actually be optimized for more targeted education, but the existing work structure limits SHUs to functioning as “mini clinics.” In the context of sensitive issues (e.g., reproductive health, body literacy, violence prevention), the potential of SHU as a provider of specific education could be significant but currently remains unrealized due to its passive and wait-and-see role.
  A2: “So perhaps the SHU staff can provide more specific education than the Guidance and Counseling staff, …”.
  A4: “What I’ve seen so far is that the SHU staff only wait in the SHU. So, for example, if a student is feeling unwell on a regular school day.”
3. Reliance on External and Informal Sources for Sexual Violence Prevention Education
  Because knowledge comes from occasional visits to community health centers and from the highly diverse internet, participants risk obtaining information that is inconsistent (between moral messages and health messages), partial/random (depending on the content that appears on their FYP), or unverified (even though there is content from professionals, the platform is still mixed with misinformation). This is based on the following statement.
  A5: “So, community health center staff come to our class to explain. First, sometimes it’s about healthy eating, then, like, the caregivers, what’s it called, they explain the consequences of extramarital sex and the women’s menstrual cycle….”
  A7: “I get this knowledge from the internet.” “Yes, because on TikTok, for example, there are a lot of midwives, teaching us about hormones.”

### Theme 2: Threats and negative consequences of sexual-social activities in the digital space

Based on statements from female adolescents, four participants had indirect experience with ESBV (through schoolmates): participants 2, 5, 6, and 7. This is supported by quotes in themes 2 and 3.

1. Perceived Health and Social Consequences of Online Interactions
  Quotes from participants indicate that excessive use of social media can be seen as a factor that increases vulnerability to relational risks. Participants’ statements regarding this are as follows:
  A3: “Like PaP (Post a Picture), or perhaps you can get to know each other through the internet, online dating, which can ultimately lead to unwanted relationships, marriage at a young age, and so on.”
  A4: “I spend too much time on social media, to the point where I forget to study, forget about daily activities, and forget to interact socially with people.”
  P: “Getting to know the opposite sex through social media platforms such as Facebook, Instagram, and WhatsApp.
2. Unfamiliar Online Contacts as a Source of Threat
  The following statements by adolescent girls.
  A1: “The risk is that with social media, we often don’t know who is behind it, so they could be pretending to be someone else.”
  A2: “So it looks like he wants to ask something, like maybe taking off my hijab or changing my outfit like that.”
  A7: “Maybe it’s from his behavior and words. If his words are rude and often suggestive, like, ‘Hey, who are you sleeping with tonight? Come with me on VC,’ like that.”
  The ambiguity of identity allows perpetrators to build interactions with fake personas, then test boundaries through requests related to appearance, and subsequently engage in verbal sexual harassment and push communication into private spaces. This shows that participants understand digital risks as an escalatory process, rather than a single incident. The second and seventh adolescent participants stated that they had almost become victims, as evidenced by their quotes, but they were able to recognize the risky behavior of online criminals.
3. Uncontrolled Circulation of Sexual Content in Social Media
  The following quote confirms that social media is understood as a mechanism that accelerates the spread of information to the point of going viral, thereby exerting a powerful social influence.
  A2: “It’s… It’s posted on social media, it’s made viral, so it can have social influence.”
  A7: “It was spread on a 9th grader’s phone yesterday. But maybe it was spread again throughout the school, and the whole school already knew about it.”
  P: “There’s already viral and all that stuff too.
  Well, the majority of the conditions when we meet are mostly unstable psychological conditions.
4. Normalization of Risky Sexual and Social Behaviors Online
  This quote shows a shift in norms: behaviors that were once taboo are now becoming normalized, both in dating and in digital practices such as exchanging inappropriate photos/videos. At the same time, intergenerational tensions are emerging: some parents still forbid dating, while others legitimize it for emotional or pragmatic reasons.
  A1: “… Also, nowadays, there’s a lot of normalization of inappropriate things. It could even include sending inappropriate photos/videos to each other.”
  A3: “…parents still have the stigma of not dating. Nowadays, many things have been normalized, perhaps one of them is dating. Some parents might even say, ‘Well, find a boyfriend, maybe to cheer you up, or maybe he can pick you up and drop you off.‘"
  However, some teenagers also have the awareness that they must be able to filter behavioral trends, as not all are good to imitate.
  A8: “Actually, it’s not that good because not all trends can be FOMO into.”

### Theme 3: Individual and social vulnerabilities contributing to electronic-based sexual

1. Individual-Level Vulnerabilities: Motivations and Patterns of Social Media Use
  The following excerpt shows that social media is used to fulfill the need for affection and validation through messages of praise, while also serving as a “window” to recognize the attitudes of people outside one’s immediate environment.
  A2: “If… through real people, for me, they don’t give attention through words, so I prefer people who text, like… you look really pretty today, like that, right?”
  A8: “But since using social media, I have begun to discover what people’s attitudes are like outside.”
2. Individual-Level Vulnerabilities: Early and Intensive Exposure to Digital Devices
  Exposure to the digital space is established long before high school age, which has the potential to influence habits, digital literacy, and ways of building relationships. quotes that support this:
  A2: “I think it was since elementary school, maybe around grade 10.”
  A6: “I’ve had my own cell phone since grade 5.”
3. Individual-Level Vulnerabilities: Low Self-Esteem and Emotional Vulnerability
  Negative social experiences in the real world (especially bullying) can encourage some teens to seek acceptance in online spaces. In online spaces, immediate judgment can be masked because identities and physical conditions are not visible.
  A1: “Maybe they feel like they don’t fit in with their real friends like they do in class because they’re usually the victims of bullying. So, they feel better making friends online, because people online can’t see what they’re really like.”
  A2:” When we talk through social media, we don’t talk face to face, so maybe they feel insecure about their physical appearance, whereas on social media, we don’t know what their face looks like or what their body looks like.
4. Individual-Level Vulnerabilities: Sexting Practices and Digital Dating Violence
  Building social relationships in the digital world is prone to escalating digital relationship risks, from private interactions shifting to transactions/commercialization by men.
  A5: “She said that every night she would make sleep calls like that.” “In my opinion, that’s not good. I mean, when we’re asleep, we don’t know what position we’re in, or maybe our clothes are lifted up. Or maybe when we’re asleep, the guy wakes up and takes a photo or screenshot while our clothes are lifted up.”
  A6: “Video calls. I don’t think it’s her boyfriend, it doesn’t seem like they’re dating, but it’s something like that.”
  A7: “Yeah, there are more than 10 videos of him like that. Her boyfriend gave it to her, and then he said if she wanted to see more, she’d have to pay, like he was selling the videos.”
  P: “Dating and all that sort of thing, then later, for example, there is sexual behavior at the beginning, and eventually it starts to be suppressed. If not, let’s try again, let’s meet again. If not, I’ll spread it around.”
5. Family and Structural Vulnerabilities: Disharmonious Family Relationships and Weak Parental Supervision
  Harmonization of family relationships and the supervisory function of adults at home influence the vulnerability of adolescent girls to OCSA.
  A3: “Lack of attention from family members, or as they call it, fatherless. They don’t get a father’s role, or they don’t get a mother’s role. So they need……”.
  A7: “There’s a lack of supervision when it comes to cell phone use, because parents are so busy.”
  P: “Inappropriate parenting styles, weaknesses, and all sorts of things, that’s how it ends up.”
6. Family and Structural Vulnerabilities: Inadequate understanding of digital safety, legal protection, and sexual and reproductive health.
  This quote shows that technology is part of everyday life, so children cannot be kept away from technology, but they need to be educated on its safe and wise use. This education includes digital literacy, understanding of the law, and reproductive health education.
  A6: “I don’t think I know that much about it, so I can’t really speak on that matter.”
  A10: “I think we need more specialized training for that, Mom.”
  P: “Well, kids these days need to be educated about the use of digitalization. Because I agree that we cannot stay away from technology.”
7. Family and Structural Vulnerabilities: Female adolescents tend to be more vulnerable than males.
  ESBV risk is not understood solely as an “individual mistake,” but as a product of relational pressures and gender structures that affect self-esteem, the need for validation, and the ways in which women gain recognition.
  A5: “This girl who doesn’t have a boyfriend will do anything to attract a guy’s attention, perhaps by undermining her self-esteem. She meets a guy online and immediately shares slightly vulgar photos.”
  P:” It’s also quite powerful, ma’am. The issue of patriarchy, those norms are still very strong in our society.”
8. Family and Structural Vulnerabilities: Social norms expect adolescents to be independent in their decision-making, even though their autonomy is not
  Children do not yet have sufficient independence to make important decisions, even though social norms often assume that at a certain age they are mature enough to make their own choices.
  P: “Children do not yet have the independence to do this (make decisions), but the prevailing norm is that at that age, many people feel that their children are mature enough, older, and should be able to make their own decisions. However, in terms of maturity, experience, and knowledge, this is not sufficient grounds for making decisions about children’s futures.”

### Theme 4: Barriers and dynamics of psychological recovery for child and adolescent victims in the context of sexual violence

This fourth theme is a case theme, where the subthemes are only obtained from the statements of clinical psychologists. This theme reflects the clinical perspective of the psychologist based on professional experience in handling ESBV cases and does not represent direct victim testimony.

1. Differences in psychological manifestations based on the age of the child
  P:” Well, I’ve found that there are some distinctive differences between teenagers and children. Children, especially those under the age of 10, don’t seem to have any obvious problems.For example, they still come to school and play as usual, and they are still cheerful as usual. But actually, there are psychological problems occurring in them.” “Well, it’s different with children aged 10–12 to late teens. They are usually more emotionally expressive. You can see some of them looking gloomy, not daring to go out. Or often posting sad statuses, which is also common. Eventually, they also close themselves off, which is also very common. So the difference lies more in their expressiveness. Teenagers tended to express sadness and depression much more frequently.
  This statement shows that ESBV’s impact on emotional disorders in early childhood affects school life, social life, and future prospects.
2. Legal interests and the many parties involved hinder ongoing psychological support for child victims.
  P:”When this case went viral, it entered the legal realm. Many parties only saw this as a problem. So, for example, they looked at the needs of the case and so on. They did not see this in the context of providing support and treatment. So, person A would ask questions, person B would ask questions, and person C would ask more questions. Well, this eventually became a challenge for us psychologists, making it difficult to limit who would influence this child. Many of the victims’ children were brought or accompanied to the psychologist by parties with legal interests. After that, there was no follow-up, which eventually became a new problem in the future.”
  This quote emphasizes that the legal process for victims can be a structural factor that hinders recovery, especially if the case goes viral, because it produces many uncoordinated interventions and shifts the focus from care to case processing.
3. Taboos and stigma mean that many cases of sexual violence go unreported and carry long-term risks.
  This statement shows that it is important to create a safe social space in the form of family support, schools, health services, and institutional responses that do not blame victims so that speakecpating up is no longer perceived as a risky act.
  P:” So sexual violence is still a taboo subject, still carries a negative stigma in society. That’s why it becomes a problem in itself, because victims don’t dare to talk about it, don’t dare to feel safe enough to speak up.”

### Theme 5: Cross-sector synergy for early self-protection education

1. Collaboration between all elements (not just education or health)
  The following participant statements indicate that ESBV is a complex issue that requires a multidisciplinary approach.
  A1:”So it’s possible that the school could collaborate with the community health center.”
  P:” social environments, social environments in the sense of neighborhood associations, village associations, village officials, or community leaders also have an important role to play.” “Next is the community health center, the police, the military, and so on.”
2. Age-appropriate education, including kindergarten/early childhood education, emphasizing decision-making and personal safety.
  Participants revealed that preventing EBC is a continuous educational process across all ages that fosters children’s self-protection skills from an early age. These skills include providing information, setting boundaries, refusing, seeking help, and reporting incidents to trusted adults.
  A3:” We strive to prevent teenagers from engaging in risky behavior, so we encourage them to develop awareness from an early age.
  P:” Education must come from all aspects, all elements, and all age levels. This education begins with kindergarten and early childhood education….about how children can make decisions for themselves. I will not be touched, I refuse, I will seek help, I will tell A and B.”
3. Families, community health centers/credible health workers, and school teachers are at the forefront of building soft skills
  P:” Family and school are very important because children spend so much time at school.” “Well, apart from family, community health centers, and trusted health workers, credibility will certainly have a strong impact when it comes to educating children or the surrounding community.”

## Discussion

To aid interpretation, the emergent themes were subsequently aligned with HPM components; this mapping was used for theoretical integration and did not function as a deductive coding framework.

### Gaps in school-based sexuality education: beyond biological knowledge

Participants described school-based sexuality education as limited and often delivered through incidental, normative messages, which led some students to seek information from social media. This demonstrates a gap in protective competencies central to comprehensive sexuality education (CSE), such as recognizing risky situations, refusing unwanted requests, setting boundaries, and seeking help. In line with UNESCO’s International Technical Guidance on Sexuality Education, CSE is intended as a structured, age-appropriate, and progressively sequenced curriculum that covers body literacy, consent, healthy relationships, digital literacy, and help-seeking strategies [40–43]. Integrating CSE within the curriculum may improve message consistency and enable more systematic program evaluation. Participants also noted the potential role of School Health Units (SHU) as accessible channels for health education, confidential consultation, and referral coordination; effective implementation would likely require staff capacity-building, whole-school engagement, communication with parents/community, and clear school-level policies [44–46].

### Threats and negative consequences of sexual-social activities in the digital space

All participants reported using the internet and social media for digital socialization. They also described digital environments as creating interconnected health and social risks that can escalate over time, including disruption to learning routines and face-to-face relationships and increased exposure to emotionally manipulative interactions such as online grooming [47, 48]. Grooming may be initiated by known or unknown contacts and is facilitated by platform characteristics such as anonymity, accessibility, rapid reach, and the permanence and propagation of shared content [47, 48]. Participants linked viral dissemination of sexual content to reputational harm and psychosocial distress within school networks. They also described a perceived normalization of risky online practices (e.g., digital dating and exchange of sexual images), shaped by peer influence and, in some cases, parental attitudes [49, 50]. Together, these observations underscore the importance of strengthening adolescents’ capacity to critically appraise online interactions and social trends [51].

### Individual and social vulnerabilities contributing to electronic-based sexual

The findings suggest that vulnerability to electronic media risks operates at two interrelated levels: individual factors and family/structural contexts. At the individual level, negative offline experiences and unmet emotional needs may increase adolescents’ motivation to seek rapid affirmation in digital environments, often through message-based praise and attention [52]. Early and sustained access to digital devices—frequently beginning in elementary school—may expose children to online socialization and relationship formation before emotional regulation and risk appraisal capacities are fully developed [53–55]. As a result, adolescents may appear “quick to trust” or “quick to become intimate” online not simply due to naivety, but because digital interactions can provide perceived safety and emotional relief [56]. Vulnerability may further intensify when interpersonal exchanges or intimate content are leveraged for coercion, blackmail, or commercialization, shifting relational dynamics toward a transactional logic [57]. In addition, the data indicate that early digital access is not consistently matched by adequate digital safety literacy. While some participants identified risks related to anonymous identities and rapid viral dissemination, they demonstrated more limited understanding of platform-based privacy controls, digital footprints and permanence, and the incremental manipulation typical of grooming processes. These variations in digital literacy appeared to shape risk interpretation: some adolescents framed potentially harmful encounters as “normal” online interactions, whereas others recognized patterns of escalating threat.

At the family and structural levels, participants described vulnerabilities that may reinforce one another. Limited supervision and unmet emotional needs were described as contributing to adolescents’ engagement in private digital relationships that are difficult for adults to monitor, creating opportunities for grooming through trust-building and emotional dependence that may later shift to control, threats, or exploitation. These accounts suggest that vulnerability is shaped not only by individual decision-making but also by gaps in guidance and support [25, 58–62]. As digital access is difficult to avoid, prevention may benefit from capacity-building in digital safety literacy, legal awareness, and sexual and reproductive health education to support risk appraisal and responses to coercion or blackmail. Participants and the key informant also linked gendered norms and patriarchal expectations to power imbalances and victim blaming, which may increase pressure on girls to seek validation and may be exploited through escalating demands [57, 63]. Finally, adolescents were sometimes perceived as autonomous decision-makers despite limited developmental readiness, which may contribute to pseudo-autonomy and reduce adult support when harms occur [64–66].

### Barriers and dynamics of psychological recovery for child and adolescent victims in the context of sexual violence

The clinical key informant noted that younger children (≤ 10 years) may show fewer observable psychological symptoms immediately after ESBV or other sexual violence, although adverse effects may emerge over time [67–69]. This highlights the importance of developmentally appropriate screening and continuity of support. Inconsistent follow-up may compromise school functioning, social relationships, and longer-term well-being. The key informant also described how fragmented responses and legally driven processes can add burden for victims when child-centred principles are not consistently applied. A supportive environment across family, school, health services, and institutions—characterized by validation, confidentiality, and non-blaming responses—may facilitate disclosure and engagement in care [70, 71]. Overall, recovery may be strengthened through coordinated, child-centred services, clear referral pathways, and stigma-reducing community responses.

### Cross-sector synergy for early self-protection education

Participants described ESBV risk as shaped by interacting influences across individual, family, peer, and school contexts, and they emphasized that prevention involves coordination among actors such as schools, families, health services, and, where relevant, child-protection and legal stakeholders, alongside community support [71–73]. Early, age-appropriate education was viewed as important for building protective skills (e.g., boundary-setting, help-seeking, and safe decision-making), with families, teachers, and trusted health workers positioned as the most immediate sources of support [74–76]. Broader multi-stakeholder approaches (e.g., pentahelix-oriented collaboration; Academics, Business, Community, Government, and Media) may provide a useful implementation frame, but their feasibility and effectiveness should be assessed in future implementation and evaluation studies.

These findings can be interpreted in relation to the Health Promotion Model [77, 78]. Individual vulnerabilities (e.g., early digital exposure, low self-esteem, and gender norms) align with personal factors and prior experiences. Adolescents’ appraisals of online interactions reflect behavior-specific cognitions, including perceived benefits (validation), perceived barriers (fear of stigma), and self-efficacy (ability to refuse and set boundaries). Peer norms, parental supervision, and school support represent interpersonal influences, while platform anonymity and rapid content dissemination represent situational influences. The expressed need for comprehensive sexuality education and digital safety training aligns with the model’s behavioral outcome component, suggesting increased commitment to protective actions. Within this framework, digital literacy may function as a cognitive resource shaping how situational exposure translates into risk appraisal and self-protective behavior. Prevention efforts may therefore benefit from structured, skills-based digital safety education (e.g., privacy management, recognizing grooming patterns, understanding content permanence, and basic legal awareness) rather than general warnings alone.

Given the local focus on Bengkulu City, transferability to other Indonesian settings should be considered cautiously. Local cultural norms may shape adolescents’ willingness to discuss sexuality and digital risk, and social desirability may have influenced some responses. In addition, the psychological recovery theme was derived from a single clinical key informant because access to victims was restricted; therefore, recovery-related findings should be interpreted as clinical insight rather than direct victim testimony.

## Conclusion

This qualitative descriptive study indicates that adolescent girls perceived gaps in school-based sexuality and digital safety education, recognized escalating online risks (e.g., anonymous contacts and rapid content circulation), and described individual and contextual vulnerabilities related to emotional needs, peer norms, and limited supervision. The clinical key informant highlighted recovery barriers and the importance of coordinated support. These findings suggest that school- and family-based interventions focusing on digital safety skills and protective communication may be beneficial in this setting. However, broader system-level policy recommendations require further implementation and evaluation research.

## Recommendation

These findings may inform locally tailored recommendations across school, family, and service settings. In schools, strengthening a systematic comprehensive sexuality education (CSE) approach may support adolescents’ protective competencies beyond biological content, including consent, boundary-setting, digital safety, and help-seeking. School Health Units (SHUs) could be strengthened as confidential entry points for consultation, early identification of digital risk, and referral pathways in coordination with school counseling services and local health facilities. At the family level, parenting programmes that enhance digital literacy and supportive communication may reduce susceptibility to online grooming and facilitate help-seeking. From a clinical perspective, the key informant highlighted the value of age-sensitive screening and child-centred care to support recovery while minimizing re-traumatization. At the system level, multi-stakeholder approaches (including pentahelix-oriented collaboration) and earlier-life prevention strategies may be considered, but their feasibility and impact require dedicated implementation and evaluation studies. Future research should consider longitudinal designs and include male adolescents to examine gendered norms and develop more inclusive prevention strategies.

## Data Availability

The data (in Indonesian) used and/or analyzed during the current study are available from the corresponding author upon reasonable request.

## Abbreviations

ESBV Electronic: Based Sexual Violence
CSE: Comprehensive sexuality education
ECPAT: End child prostitution, child pornography and trafficking of children for sexual purposes
FoMO: Fear of Missing Out
ITGSE: International technical guidance on sexuality education
OCSEA: Online Child Sexual Exploitation and Abuse
SHU: School Health Units
